# Effect of Sintering Temperatures on Grain Coarsening Behaviors and Mechanical Properties of W-NiTi Heavy Tungsten Alloys

**DOI:** 10.3390/ma15228035

**Published:** 2022-11-14

**Authors:** Yang Shao, Weikang Yu, Jifei Wu, Haiwen Ma

**Affiliations:** Department of Materials Science and Engineering, Dalian Maritime University, Dalian 116026, China

**Keywords:** NiTi binder, tungsten heavy alloys, activation energy

## Abstract

W-NiTi tungsten heavy alloys were prepared by an infiltration process using submicron W powders, and the effect of sintering temperatures on grain-coarsening behaviors and the mechanical properties of W-NiTi tungsten heavy alloys were investigated. The microstructures and mechanical properties were investigated using scanning electron microscopy, X-ray diffraction and compression tests. The results showed that tungsten particles were uniformly distributed in the NiTi binder. The W-NiTi tungsten heavy alloys consisted of B19′-NiTi and body-centered cubic W phases. The average tungsten particle sizes of W-NiTi tungsten heavy alloys sintered at 1400 °C, 1480 °C and 1560 °C were 2.62 μm, 4.04 μm and 5.20 μm, respectively. The average tungsten particle size increased with sintering temperatures, while the densities decreased at higher temperatures. The cavities retained in the W-NiTi tungsten heavy alloy sintered at 1560 °C, which degraded the mechanical properties. The calculated grain growth activation energy of W particles in the NiTi binder was 330 kJ/mol, which was higher than those in conventional W-NiFe and W-NiCo tungsten heavy alloys. The higher activation energy means more difficult diffusion process of W atoms in NiTi binders during sintering. Therefore, finer-grained heavy tungsten alloys were more easily obtained by using NiTi binders. Yield strength of W-NiTi tungsten heavy alloys decreased with increasing sintering temperatures due to coarsened tungsten particles.

## 1. Introduction

Tungsten heavy alloys (WHAs) have been widely used in aerospace, military, and electronics industrial fields due to their high density, high conductivity and excellent mechanical properties [1,2]. WHAs usually consist of body-centered cubic (BCC) tungsten (W) particles and face-centered cubic (FCC) binder phases, such as NiFe [3], NiCu [4], NiCo [5] and so on. Common WHAs are usually prepared using liquid phase sintering at high temperatures above 1460 °C [6,7]. Tungsten particles coarsen severely during liquid phase sintering due to Ostwald ripening [7]. The large tungsten particle size degrade the mechanical properties of WHAs and restrict their application.

Several methods have previously been applied to refine tungsten grain size in WHAs [8,9,10,11,12,13]. Some advanced sintering methods have been used to prepare fine-grained WHAs, such as two-step sintering [8], solid state sintering [9], spark plasma sintering [10], etc. Alloying with some elements is another method [11]. It has been reported that Mo addition could reduce the solubility of W in matrix and retain W grain coarsening during sintering in W-Ni-Fe-Mo WHAs [11]. Based on previous reports [11,12,13,14], the matrix has a strong impact on W grain-coarsening behavior, so explorations of new matrices is an effective way to refine W particle size. Recently, a novel WHA consisting of W and NiTi shape memory alloys has been prepared using infiltration and hot pressing [13]. It has been reported that the W particle was very stable in NiTi shape memory alloy matrix during infiltration. However, the effect of sintering temperatures on W grain coarsening behaviors has not been investigated in W-NiTi WHAs during sintering.

In this paper, W-NiTi WHAs were prepared with sub-micrometer W powders using infiltration. The effect of sintering temperatures on the W grain coarsening behavior was investigated in detail. 

## 2. Materials and Methods

**Figure 1:**
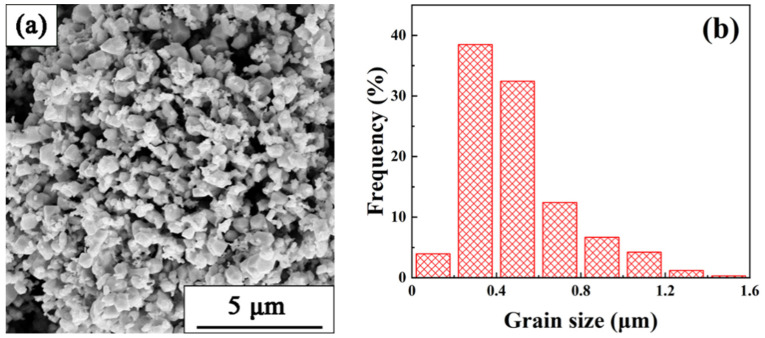
(**a**) The morphology and (**b**) power size distribution of raw W powders.

**Figure 2:**
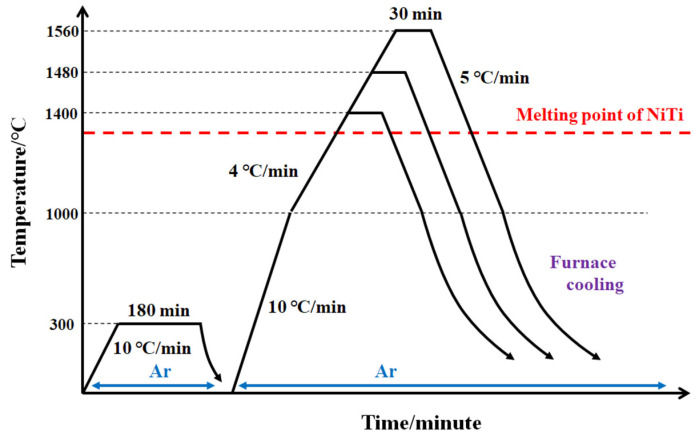
Detailed sintering process of W-NiTi WHAs.

W powders with an average particle size of 494 nm used in this study were purchased from Chengdu Huayin powder technology Co., Ltd. (Chengdu, China). The morphologies and distributions of particle sizes of the raw W powders are shown in Figure 1. An alloy with composition of Ti_53_Ni_42_Nb_5_ (at.%) was prepared using arc melting under pure argon atmosphere. The composition design of the master alloy has been reported in a previous study [15]. The W powders were mixed with octadecanoic acid alcohol solution at 60 °C. Then, the mixture of octadecanoic acid and W powders was obtained after the evaporation of alcohol. The content of octadecanoic acid in the mixture was 1.5% in weight. Then, the mixture was pressed into blocks with a dimension of ∅30 mm under a pressure of 150 MPa. The blocks were heated in a tubular sintering furnace under argon atmosphere at 300 °C for 3 h to evaporate octadecanoic acid. Ti_53_Ni_42_Nb_5_ alloy was then put on the blocks. The infiltration processes were conducted at different temperatures (1400 °C, 1480 °C, 1560 °C) and holding for 30 min in a graphite mold under argon atmosphere in tubular sintering furnace. The detail sintering process is shown in Figure 2.

Densities of sintered WHAs were determined using Archimedes’ method. Microstructures of sintered WHAs were examined using a scanning electron microscope (SEM, Supra 55 Sapphire, Carl Zeiss, Jena, Germany). Phase constitutions were obtained using X-ray diffraction (XRD, Rigaku SmartLab X-ray diffractometer, Tokyo, Japan) using Cu *Kα* radiation. The compression mechanical properties of W-NiTi WHAs were conducted using a universal mechanical test with a speed of 0.5 mm/min at room temperature. The specimen for compression was cut with wire cutters with a dimension of ∅3 × 6 mm.

## 3. Results and Discussions

**Figure 3:**
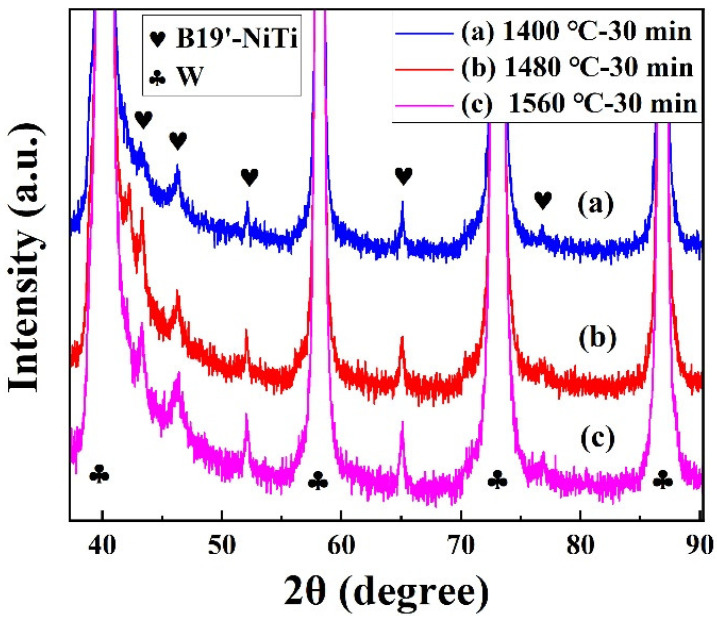
XRD patterns of W-NiTi heavy tungsten alloys.

Phase constitutions of W-NiTi heavy tungsten alloys infiltrated at different temperatures are shown in Figure 3. The strongest diffraction peaks in all samples were in the W phase, while the weak peaks corresponded to B19′-NiTi. From Figure 3, all W-NiTi heavy tungsten alloys consisted of B19′-NiTi and W phases. The results were consistent with previous reports [15].

**Figure 4:**
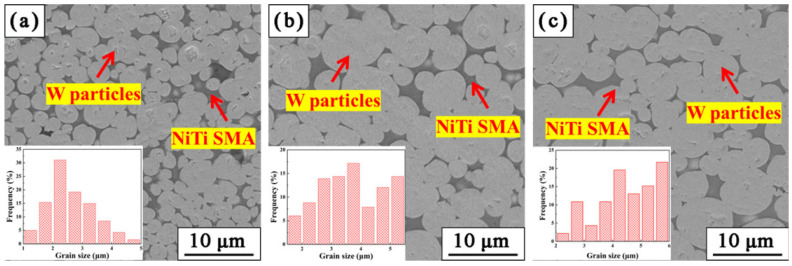
(**a**–**c**) Microstructures of W-NiTi sintered at different temperatures: (**a**) 1400 °C; (**b**) 1480 °C; (**c**) 1560 °C.

**Figure 5:**
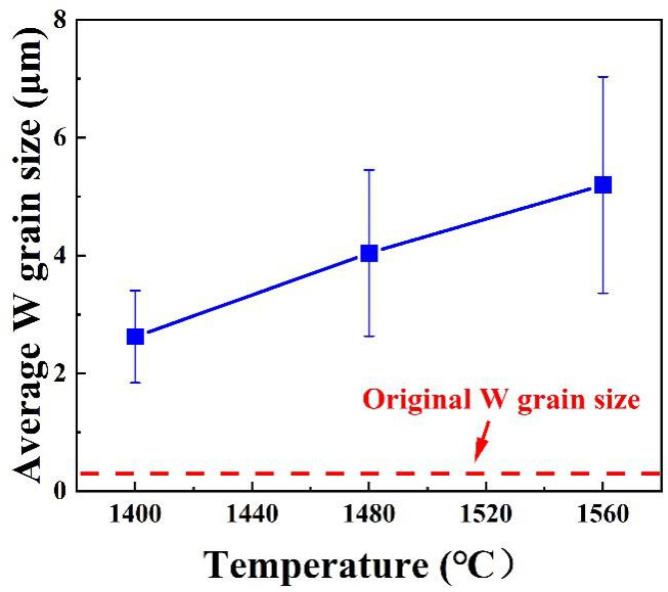
Evolutions of average W particle sizes of W-NiTi WHAs with sintering temperatures.

The microstructures of W-NiTi WHAs sintered at 1400 °C, 1480 °C and 1560 °C are shown in Figure 4a–c. It can be seen that tungsten particles uniformly dispersed in the NiTi matrix. The polygonal tungsten particles, as shown in Figure 1, grew into nearly spherical shapes. W particle sizes of the WHAs are summarized in Figure 5. It is obvious that the average W particle sizes in W-NiTi tungsten heavy alloys were 2.62 μm, 4.04 μm and 5.20 μm, respectively. The average W particle sizes were larger than that of raw W powders (~494 nm.). Therefore, W particles coarsened during the sintering process. Meanwhile, W particle size increased gradually with increasing sintering temperatures.

According to a previous report [7], W-particle-coarsening behavior during liquid phase sintering was mainly due to Ostwald ripening and was controlled by the diffusion of W atoms. Activation energy of W grain growth in NiTi can be calculated using the following equations [16]:

*D*^3^−*D*_0_^3^ = *Kt* (1)

**Figure 6:**
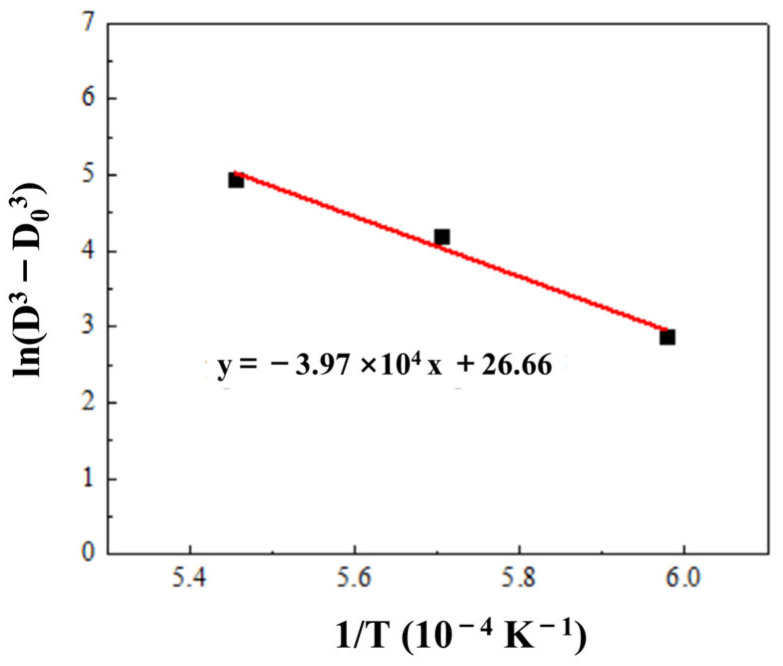
Relationship between *ln*(*D*^3^*−D*_0_^3^) and 1/*T*.

**Figure 7:**
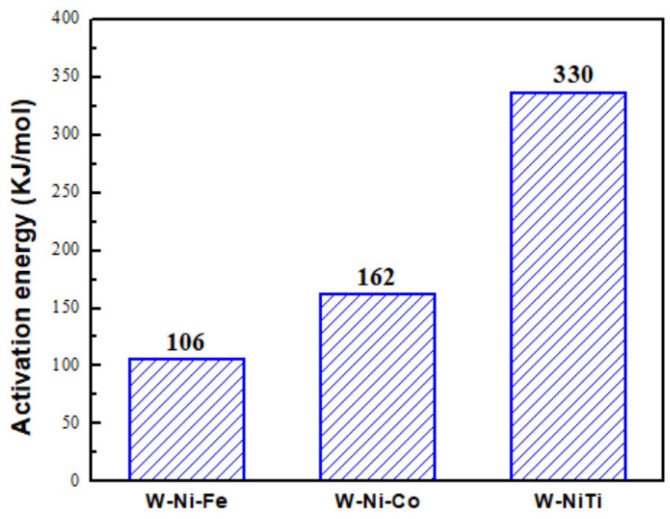
Comparisons of activation energies of W particles in NiTi and other binders [16,17].

*K* = *K*_0_*exp(*−*Q/RT)* (2)

where *D*, *D*_0_, *K*, and *t* are the average W particle size of WHAs, the average particle size of raw tungsten powders (494 nm), and a parameter dependent on temperatures and holding time during infiltration (1800 s), respectively. *Q* is the activation energy of W particles in binders. *R* and *K*_0_ are constant, T is the absolute temperature. The activation energies can be derived from the slope of a curve for *ln*(*D*^3^*−D*_0_^3^) to (1/*T*), as shown in Figure 6. The calculated activation energy of W in NiTi binder was calculated to be about 330 kJ/mol, which was higher than those in conventional NiCo and NiFe binders [16,17]. Figure 7 depicted comparisons of the activation energies in different binders.

The higher activation energy means a more difficult diffusion process of W atoms during sintering. The results suggested that W grain growth in the NiTi binder was suppressed during liquid phase sintering. Therefore, tungsten heavy alloys with finer W grains could be easily obtained by using a NiTi binder [13].

**Figure 8:**
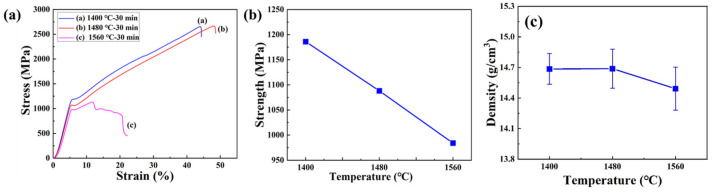
Mechanical properties of W-NiTi heavy tungsten alloys under compression tests at room temperature. (**a**) Stress–strain curves under compression tests. (**b**) Relationship between yield strength and density with sintering temperatures. (**c**) The relationship of actual density of W-NiTi tungsten heavy alloys with infiltration temperature.

The densities of W-NiTi heavy tungsten alloys, measured by Archimedes’ method, are shown in Figure 8c. From this figure, the densities of W-NiTi tungsten heavy alloys sintered at 1400 °C, 1480 °C, and 1560 °C were 14.69 g/cm^3^, 14.68 g/cm^3^, and 14.51 g/cm^3^, respectively. The densities of W-NiTi heavy tungsten alloys decreased gradually with increasing infiltration temperatures. The density of WHA sintered at 1560 °C was the lowest, which indicated some pores were retained in this alloy. A similar phenomenon was also reported in W-NiFe WHAs [18,19]. This could be attributed to the trapped gases within the residual pores in tungsten blocks. The pressure of trapped gases in pores expands and impedes liquid-phase to fill them at higher temperatures. As a result of this, pores remained in the alloy, leading to lower densities. 

The stress–strain curves under compression tests of W-NiTi heavy tungsten alloys at room temperature are given in Figure 8a. Failure strain of W-NiTi heavy tungsten alloy infiltrated at 1400 °C was ~44%, which was slightly lower than that sintered at 1480 °C, while for WHA sintered at 1560 °C, it failed at a strain of ~11.7%, much lower than others, which possibly resulted from the pores remaining in W-NiTi heavy alloys. The W-NiTi WHAs sintered at 1400 °C exhibited the highest yield strength of 1186 MPa. Yield strength of W-NiTi WHAs decreased gradually with sintering temperatures, which was because of coarsened W particles at high temperatures (Figure 4).

**Figure 9:**
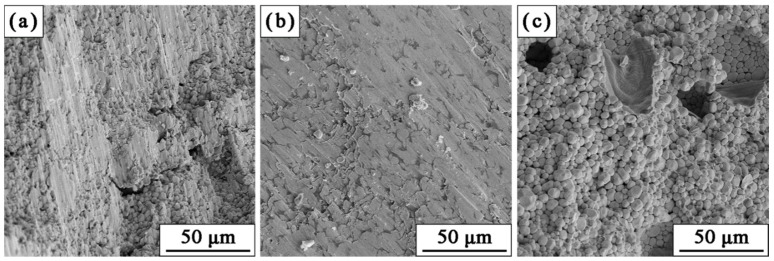
Fracture morphologies of W-NiTi WHAs sintered at different temperatures after compression tests. (**a**) 1400 °C; (**b**) 1480 °C; (**c**) 1560 °C.

To further investigate the deformation behavior under compression, fracture microstructures of W-NiTi tungsten heavy alloys were examined, as shown in Figure 9. After compression tests, the fracture surfaces were relatively smooth for W-NiTi WHAs sintered at 1400 °C and 1480 °C, which may result from the friction between two fractured parts of samples during compression. Furthermore, almost all of W particles fractured in cleavage mode. W-W intergranular fracture and W-matrix fracture were not observed from fractured surfaces. However, for WHA sintered at 1560 °C, the fracture surface was relatively coarse. Meanwhile, many cavities can be seen in this alloy, which was possibly because the trapped gases impeded the liquid-phase to fill them at a higher temperature. The cavities formed in this alloy and thus severely deteriorated its mechanical properties.

## 4. Conclusions

W-NiTi WHAs were prepared by sintering at different temperatures using submicron W powders. The effect of infiltration temperatures on microstructures and mechanical properties of W-NiTi heavy tungsten alloys was investigated. The tungsten particles uniformly distributed in NiTi binders. The average W particle size increased from 2.62 μm to 5.20 μm. The activation energy of W particles coarsening in NiTi binders during sintering was 330 kJ/mol, which was much higher than those in conventional binders. Finer-grained WHAs were more easily obtained using NiTi binders. The density and yield strength decreased gradually with increasing sintering temperatures. W particle coarsening resulted in decreased yield strength. The cavities formed in W-NiTi WHA sintered at a higher temperature damaged the mechanical properties.

## Data Availability

All relevant data are available upon request from the authors.
